# T Cell Epitope Discovery in the Context of Distinct and Unique Indigenous HLA Profiles

**DOI:** 10.3389/fimmu.2022.812393

**Published:** 2022-05-06

**Authors:** Luca Hensen, Patricia T. Illing, Louise C. Rowntree, Jane Davies, Adrian Miller, Steven Y. C. Tong, Jennifer R. Habel, Carolien E. van de Sandt, Katie L. Flanagan, Anthony W. Purcell, Katherine Kedzierska, E. Bridie Clemens

**Affiliations:** ^1^Department of Microbiology and Immunology, University of Melbourne, at the Peter Doherty Institute for Infection and Immunity, Parkville, VIC, Australia; ^2^Department of Biochemistry and Molecular Biology & Infection and Immunity Program, Biomedicine Discovery Institute, Monash University, Clayton, VIC, Australia; ^3^Menzies School of Health Research, Darwin, NT, Australia; ^4^Indigenous Engagement, CQUniversity, Townsville, QLD, Australia; ^5^Victorian Infectious Diseases Service, The Royal Melbourne Hospital at the Peter Doherty Institute for Infection and Immunity, Melbourne, VIC, Australia; ^6^Department of Infectious Diseases, The University of Melbourne at the Peter Doherty Institute for Infection and Immunity, Melbourne, VIC, Australia; ^7^Department of Hematopoiesis, Sanquin Research and Landsteiner Laboratory, Amsterdam UMC, University of Amsterdam, Amsterdam, Netherlands; ^8^Department of Infectious Diseases and Tasmanian Vaccine Trial Centre, Launceston General Hospital, Launceston, TAS, Australia; ^9^School of Health Sciences and School of Medicine, University of Tasmania, Launceston, TAS, Australia; ^10^Department of Immunology and Pathology, Monash University, Melbourne, VIC, Australia; ^11^School of Health and Biomedical Science, RMIT University, Melbourne, VIC, Australia

**Keywords:** CD8^+^ T cell epitopes, Human Leucocyte Antigen (HLA), influenza, SARS-CoV-2, Indigenous populations, epitope discovery, antigen presentation, immunopeptidome

## Abstract

CD8^+^ T cells are a pivotal part of the immune response to viruses, playing a key role in disease outcome and providing long-lasting immunity to conserved pathogen epitopes. Understanding CD8^+^ T cell immunity in humans is complex due to CD8^+^ T cell restriction by highly polymorphic Human Leukocyte Antigen (HLA) proteins, requiring T cell epitopes to be defined for different HLA allotypes across different ethnicities. Here we evaluate strategies that have been developed to facilitate epitope identification and study immunogenic T cell responses. We describe an immunopeptidomics approach to sequence HLA-bound peptides presented on virus-infected cells by liquid chromatography with tandem mass spectrometry (LC-MS/MS). Using antigen presenting cell lines that stably express the HLA alleles characteristic of Indigenous Australians, this approach has been successfully used to comprehensively identify influenza-specific CD8^+^ T cell epitopes restricted by HLA allotypes predominant in Indigenous Australians, including HLA-A*24:02 and HLA-A*11:01. This is an essential step in ensuring high vaccine coverage and efficacy in Indigenous populations globally, known to be at high risk from influenza disease and other respiratory infections.

## Introduction

Novel respiratory viruses pose a major global pandemic threat associated with high disease burden and mortality. To date, the 2019 SARS-CoV-2 virus has caused more than 470 million confirmed cases and more than 6.1 million deaths reported to WHO across the world (1). Likewise, influenza viruses are responsible for annual seasonal epidemics that cause 243,000-645,000 deaths (2) and sporadic pandemics, such as the 1918-1919 H1N1 pandemic, which lead to an estimated 50 million deaths worldwide (3, 4). Outbreaks of the avian-derived H7N9 and H5N1 viruses have been associated with high rates of hospitalisation (both >99%) and death (39% H7N9 and 56% H5N1 case fatality ratio (CFR) (5)), highlighting the pandemic potential of these emerging novel influenza viruses. Indigenous populations worldwide are disproportionately affected by greater infection rates, disease severity and mortality when novel pandemic virus strains enter human circulation. During the 1918-1919 H1N1 influenza pandemic, higher rates of mortality were reported amongst the Māori people of New Zealand (7-fold higher than the European population (6)), First Nations people of Canada (8-fold higher than non-First Nations (7)), Alaskan Natives (100% of adults in some populations (8)), Indigenous Australians (10-20% compared to <1% of other Australians (9)) and Western Samoans (19-22% of total population (10)). Although less severe, the 2009 H1N1 pandemic virus also caused higher hospitalisation and mortality rates for Indigenous Australians (6.6-fold higher hospitalisation and 5.2-fold higher mortality than Non-Indigenous Australians (11)), New Zealand Māori (5-fold higher hospitalization (12)), Pacific Islanders (7-fold higher hospitalization (12, 13)), American Indians and Alaskan Natives (4-fold higher mortality (14)) and First Nations people of Canada (3-fold higher hospitalisation (15)). Emerging evidence from the UK and USA suggests a possible association between ethnicity and mortality due to SARS-CoV-2 infection (16–19), with mortality rates 3.3 times higher for Indigenous Americans compared to White Americans (20), though more global data are needed that specifically consider Indigenous populations. In the Brazilian Amazon region, the combination of SARS-CoV-2, an immunologically vulnerable Indigenous population and limited healthcare facilities has been associated with disproportionate case numbers and lethality rates among Indigenous people (21, 22). It is important to acknowledge the roles of socioeconomic disparity, high rates of comorbidities, reduced access to health services, occupation and household/community/environmental characteristics in contributing to higher rates of exposure and disease severity in ethnic minorities and Indigenous populations (16–19, 23, 24). However, underlying hereditary host and immunological factors may also contribute, including HLA genotype and polymorphisms in angiotensin-converting enzyme 2 (ACE2), transmembrane protease serine-type 2 (TMPRSS2) and interferon-induced transmembrane protein 3 (IFITM3) genes (reviewed in (25–27)). With the continuing threat of emerging respiratory viruses, targeted measures to boost immunity through vaccines, particularly those that activate long-lasting, cross-strain protective CD8^+^ T cells offer a means of protecting high risk groups, including the ~370-500 million Indigenous Peoples worldwide (28).

T cells play a key role in the resolution and modulation of disease severity in acute respiratory virus infections such as influenza and SARS-CoV-2. Mild to moderate influenza and SARS-CoV-2 infections are associated with prototypical antiviral immune responses involving transient, co-ordinated activation and contraction of virus-specific CD4^+^ and CD8^+^ T cells, B cells and antibodies (29–32). Meanwhile, severe SARS-CoV-2 infection is associated with dysregulation of cytokines and ligands (IL-18 and sIL-6R), hyperactivation of innate and adaptive immune cell compartments (marked by CD38 and HLA-DR expression), high SARS-CoV-2-specific antibody titres as well delayed SARS-CoV-2-specific CD4^+^ and CD8^+^ T cell responses (31, 32). Similarly, severe influenza disease is characterised by increased inflammatory cytokines and diminished influenza-specific responses from CD8^+^ T cells, CD4^+^ T cells, NK cells, MAIT cells, γδ T cells and antibody secreting cells (ASC) (33). While antibody-based vaccines targeting surface glycoproteins (e.g. Spike protein (S) of SARS-CoV-2 and hemagglutinin (HA) and neuraminidase (NA) of influenza) are an effective way to combat infection, potentially providing sterilizing immunity, they can fail to provide protection when antigenically different strains of virus emerge. These new strains can arise through gradual changes in the antigenicity of viral proteins, driven by rapid virus mutation and immune selection of viral mutants that can evade antibody binding (antigenic drift), or *via* recombination of viral genomes, resulting in a substantially new virus that escapes pre-existing antibody responses (antigenic shift) (34). This is seen during influenza pandemics (antigenic shift) and epidemics (antigenic drift) and now with emerging SARS-CoV-2 variants that are less susceptible to natural and vaccine-induced antibody responses (35). The unpredictable and evolving nature of these acute respiratory viruses undermines the efficacy of existing antibody-based vaccines and necessitates the annual reformulation of influenza vaccines to protect against unpredictable emerging strains, with varying efficacy (36). In contrast to antibody immunity, T cell immunity is mediated by CD8^+^ T cells that lyse virus-infected cells expressing Class I Major Histocompatibility Complex (MHC-I) molecules presenting virus peptides and CD4^+^ T cells that recognise Class II MHC (MHC-II) molecules presenting virus peptides and can kill infected cells or play a role in co-ordinating the immune response, including B cell responses and memory responses. Unlike neutralizing antibodies, the antigenic targets of T cells can be peptide+MHC epitopes derived from internal virus proteins that are often functionally significant and more highly conserved across strains, providing the basis for heterologous or “universal” immunity across unrelated strains (30, 37, 38). In situations where new viruses emerge that evade existing antibody responses, the recall of cross-reactive memory T cells that provide broadly heterotypic protection can reduce the severity of infection. This was demonstrated during the 2013 H7N9 influenza outbreak, where a shorter recovery time from severe H7N9 disease was associated with early and robust CD8^+^ T cell responses, and prolonged hospital stays with late recruitment of CD4^+^ and CD8^+^ T cells (39). Thus, vaccines that activate cross-strain protective cellular immunity from T cells represent an effective means of protecting against the threat of unpredictable and evolving acute respiratory viruses.

Human MHC, known as Human Leukocyte Antigen (HLA) molecules, display a high degree of polymorphism in the residues that line the peptide-binding pocket, which influences the array of peptides that can be bound in the pocket based on favourable peptide-HLA interactions. Different HLA alleles will bind different peptides which can often be defined by characteristic motifs compatible for binding. Indigenous populations express distinct HLA profiles that differ from the profiles of other ethnic groups. For instance, HLA-A*34:01 (30%), A*24:02 (24%) and B*13:01 (24%) are among the most common HLA Class I (HLA-I) alleles expressed by Indigenous Australians (40, 41) and, while shared to some extent with other Indigenous populations (often in geographic proximity), these are found at lower frequencies at a global population level (A*34:01 0.3%, A*24:02 10% and B*13:01 0.6%) (Allele Frequency Net Database (42)). Other alleles such as A*24:06, A*24:13 and HLA-B*56:56 are uniquely described in Indigenous Australians, though at low frequency (<1%) (40). Thus, the CD8^+^ T cell responses of Indigenous Australians are likely to target very distinct peptide epitopes compared to other ethnicities, a key consideration for the design and evaluation of T cell vaccines that can provide coverage and efficacy across ethnically diverse global populations. This is highlighted by the finding that some current influenza vaccine candidates lack key components for immunogenicity in HLA-A*24:02^+^ individuals (41). HLA-A*24:02 is associated with risk of severe influenza disease (43) and notably is expressed by a significant proportion of Indigenous Australians (24%) (40, 41) and Alaskans (58%) (42). Whilst CD8^+^ T cell responses to IAV typically focus on epitopes derived from the virus Nucleoprotein (NP), Polymerase basic subunit B1 (PB1) and Matrix protein 1 (M1) (30, 37, 44, 45), immunogenic HLA-A*24:02-restricted IAV epitopes are derived from PB1 and Polymerase basic subunit B2 (PB2) proteins (41), representing a different focus in the viral proteins targeted. HLA-A*68:01, which is highly expressed in Indigenous populations from Alaska (15% among Alaskan Yup’ik) (46) and Southern and Central America (range 2.5%-61.54%, median 12% among Amerindians) (42), was also associated with severe influenza disease during the 2009 H1N1 influenza pandemic (43, 47). This may be linked to difficulties in the recruitment of CD8^+^ T cells specific for the HLA-A*68:01-NP_145-156_ epitope during IAV infection and could potentially be overcome with repeated boosting (48).

Associations between HLA genotype and disease severity are seen for several viruses including human immunodeficiency virus 1 (HIV-1) (49, 50), dengue (51, 52), human papilloma virus (HPV) (53), hepatitis C virus (HCV) (54, 55) and SARS-CoV-1 (56) (reviewed in (57)). Likewise, certain alleles are associated with risk of tuberculosis infection (58), susceptibility to type 1 Diabetes (59, 60) or increased risk of developing autoimmune responses directed, for instance, to myelin (61) or dietary antigens (62). For influenza, five HLA-I alleles (A*02:01, A*03:01, B*57:01, B*18:01 and B*08:01) are linked with robust, cross-protective CD8^+^ T cell responses against all human influenza A viruses (63). Conversely, the aforementioned A*24:02 and A*68:01 alleles are associated with increased mortality to the 2009 H1N1 virus (43) and diminished (41) or poorly recruited (48) memory CD8^+^ T cell populations in uninfected donors, respectively. In a cohort of healthy individuals who received a seasonal influenza A vaccine, HLA-DRB1*11:04, DRB1*16:01, DQB1*05:02 and DPA*02:02 were marginally associated with higher antibody titres, while DRB1*13:03 was associated with lower antibody titre post vaccination (64). Emerging data indicate that expression of certain HLA alleles may also influence the outcome of SARS-CoV-2 infection (65–67), though this association is not seen in other studies (68, 69) (reviewed in (70)). Using HLA frequency data and *in silico* HLA-binding predictions applied across the SARS-CoV-2 proteome, HLA-B*15:03 (most highly expressed in Sub-Saharan African populations (42)) showed the greatest capacity to present SARS-CoV-2 peptides that are highly conserved among other pathogenic human coronaviruses (66). Although not validated experimentally in human donors, the findings suggest that expression of HLA-B*15:03 is associated with broadly protective CD8^+^ T cell immunity to SARS-CoV-2. Meanwhile, HLA-B*46:01 (most highly expressed in South-East Asian populations (42)) was predicted to bind the fewest SARS-CoV-2 peptides (66), suggesting diminished capacity for CD8^+^ T cell responses and increased susceptibility to severe disease, as observed in SARS-CoV-1 (56). Given that HLA profiles are heritable and influenced by ethnicity, such HLA-related effects on adaptive immunity may have greater impact on populations with distinct or highly restricted HLA profiles, such as Indigenous populations. For instance, the five HLA-I alleles associated with “universal” immunity to IAVs are reasonably common in Caucasian populations (57%), but less so in Indigenous Alaskans and Australians (both 16%) (63) which are populations with a history of disproportionate influenza disease.

Thus, to avoid potential immunological gaps and optimize protective immunity across different Indigenous groups, the development and evaluation of T cell-activating vaccines may benefit from consideration of the particular HLA expressed and the specific array of peptides they present during infection. Whilst vaccine approaches are logically aimed at achieving broad coverage of global populations, either through inclusion of large epitope rich portions of the target pathogen (71, 72) or polyepitopes (41), this may result in exclusion of the best epitope candidates for some HLA profiles, potentially impacting vaccine efficacy in some populations. Tailoring vaccines based on population HLA profiles could theoretically help achieve optimal immunity and protection in these situations, yet implementing this across the HLA diverse human population on a global scale currently poses significant challenges. Broadening our understanding of immunologically relevant epitopes for Indigenous populations can potentially guide the search for optimal vaccine targets, ensure wide population coverage of vaccines and form the basis for evaluating vaccine responses which can then inform the next generation of vaccines, vaccine regimens (adjuvanting/dose frequency/high dose), development of public health measures and use of immunotherapies.

**Figure 1:**
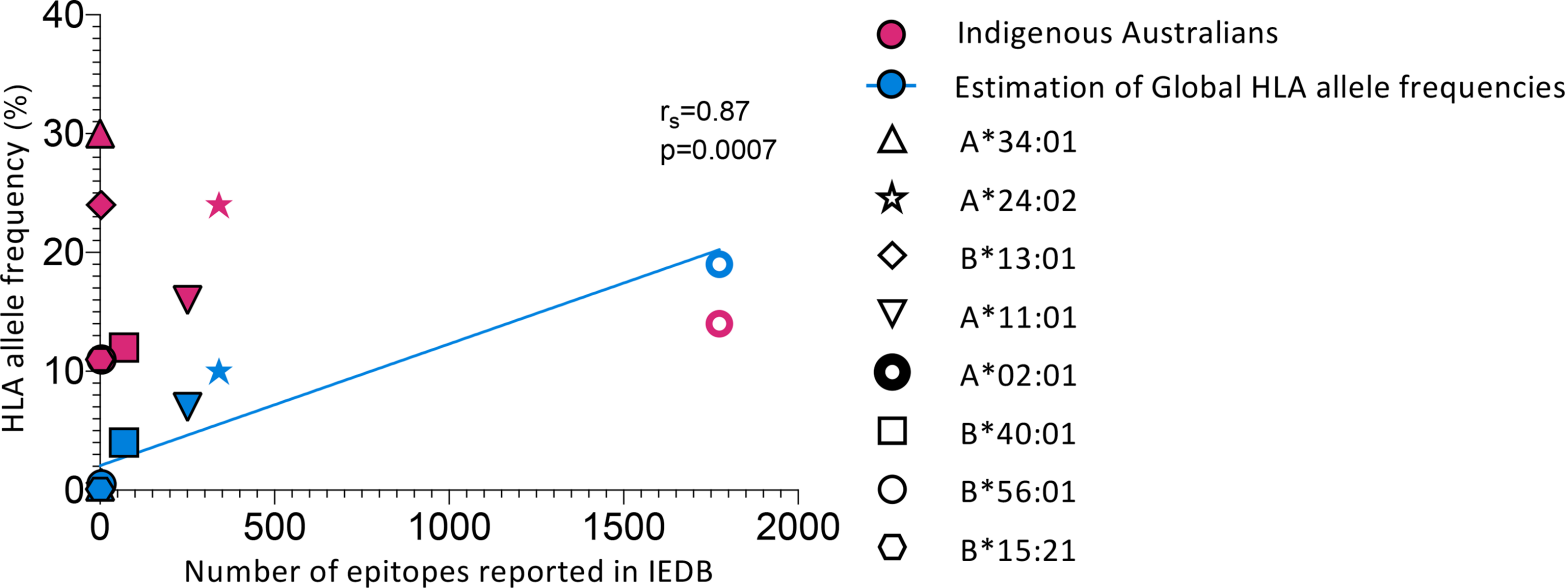
Indigenous Australian HLA alleles are underrepresented in epitope discovery. Comparing HLA allele frequencies from global population estimates (42) and Indigenous Australians (LIFT cohort) (40) with the number of epitopes reported in the Immune Epitope Database (IEDB) (73). Search filters for reported epitopes in the IEDB were: Epitope : Any, Host : Human, Assay:T cell, MHC restriction:Specific alleles indicated in the figure (HLA-A*34:01, A*24:02, B*13:01, A*11:01, A*02:01, B*40:01, B*56:01 and B*15:21), Disease : Any. Only epitopes from organisms other than *homo sapiens* were counted. See **Table 1** for full details of organisms and number of epitopes reported for each allele considered (except HLA-A*02:01). Blue line shows correlation (Spearman’s test) of estimates of global HLA allele frequencies and reported epitopes. HLA-A*34:01, A*24:02, B*13:01, A*11:01, A*02:01, B*40:01, B*56:01 and B*15:21 represent the eight most common (>10%) HLA-I alleles expressed by Indigenous Australians (40).

The identification and study of CD8^+^ T cell epitopes has traditionally focused on common and widely-expressed HLA alleles, such as HLA-A*02:01, which is highly prevalent in Caucasian populations (26%) (42). However, these well-studied HLA alleles are not always representative of ethnically diverse populations and are often found at lower frequencies in Indigenous populations. In addition, these studies may focus on one or a few immunodominant epitopes rather than comprehensively mapping the hierarchy of immunogenic epitope specificities for a given HLA. Overall, the HLA alleles most prevalent in Indigenous Australians are under-represented in epitope discovery, which typically reflects a focus on globally frequent HLA alleles (**Figure 1** and **Table 1**). There are currently no epitopes from any pathogen reported in the Immune Epitope Database (IEDB) (73) for HLA-A*34:01, the most prominent HLA allele expressed by ~30% of Indigenous Australians (up to 68% in some populations (74)) (**Figure 1** and **Table 1**). Of the eight most common (>10%) HLA I alleles expressed by Indigenous Australians, only the four most globally frequent (HLA-A*02:01, A*24:02, A*11:01 and B*40:01) currently have T cell epitopes reported for influenza A virus in the IEBD, while five (HLA-A*02:01, A*24:02, B*13:01, A*11:01 and B*40:01) have epitopes from SARS-CoV-2 reported (**Table 1**) (41). Comprehensively defining CD8^+^ T cell epitope specificities for a set of HLAs representative of Indigenous populations could assist the rational design and evaluation of vaccines that include suitable antigenic targets for priming relevant CD8^+^ T cells. Furthermore, understanding the breadth (number) and hierarchy of epitope-specific responses for these HLA allotypes is important to avoid vaccine-concentrated immune pressure on one or two epitopes leading to viral escape (75, 76). Epitope change in the presence of CD8^+^ T cell immune pressure has been observed for influenza virus in a mouse model, where mutations at anchor sites and TCR contacts for CD8^+^ T cell epitopes were identified and observed to revert in the absence of epitope-specific immune pressure (76). Furthermore, anchor mutations have been identified in the HLA-B*27:05- and B*08:01-restricted NP_383–391/380–388_ epitope that abrogate presentation and enable escape from T cell recognition (77, 78), indicating that T cell-mediated antigenic drift (44, 79) could subvert the efficacy of a T cell-based vaccine that concentrates on only a few epitopes in populations with limited HLA diversity. Comprehensively defining CD8^+^ T cell epitopes also enables consideration of how epitope-specific CD8^+^ T cell response hierarchies and immunodominance for a given HLA are influenced by HLA co-expression, cross-reactivity and infection history, which may be relevant to vaccine antigen selection and uncover associations between HLA expression and disease protection versus susceptibility.

**Table 1:** Indigenous Australian HLA alleles are underrepresented in T cell epitope discovery.

HLA allele^a^	HLA frequencyIndigenous Australians (Global)^b^	Pathogen	# Reported epitopes in IEDB^c^
HLA-A*34:01	**30%** (0.3%)	–	–
HLA-A*24:02	**24%** (10%)	Alphapapillomavirus 7 (Human papillomavirus-18)	2
		Alphapapillomavirus 9	8
		Dengue virus	36
		*Francisella tularensis*	6
		Hepatitis B virus	26
		Hepatitis C virus	41
		Human herpesvirus 1	10
		Human herpesvirus 4 (Epstein Barr virus)	23
		Human herpesvirus 5 (Human cytomegalovirus)	24
		Human immunodeficiency virus 1	11
		Human mastadenovirus B (Human adenovirus B)	3
		Human mastadenovirus C (Human adenovirus C)	1
		Human polyomavirus 1 (BK polyomavirus)	1
		Human polyomavirus 5	1
		Influenza A virus	13
		*Mycobacterium tuberculosis*	12
		Norwalk virus (Norwalk calicivirus)	1
		*Plasmodium falciparum*	1
		Primate erythroparvovirus 1	1
		Primate T-lymphotropic virus 1	8
		Rhinovirus A (Human rhinovirus A)	1
		SARS-CoV-1	1
		SARS-CoV-2	98
		*Streptococcus pyogenes*	6
		Vaccinia virus	2
		Yellow fever virus (Flavivirus febricis)	3
		Zaire Ebola virus	1
HLA-B*13:01	**24%** (0.6%)	Human herpesvirus 4 (Epstein Barr virus)	1
		Hepatitis B virus (hepatitis B virus (HBV))	1
		SARS-CoV-2	1
HLA-A*11:01	**16%** (7%)	Alphapapillomavirus 7 (Human papillomavirus-18)	2
		Alphapapillomavirus 9	7
		Dengue virus	40
		Hepatitis B virus	30
		Hepatitis C virus	6
		Human herpesvirus 4 (Epstein Barr virus)	15
		Human herpesvirus 5 (Human cytomegalovirus)	6
		Human immunodeficiency virus 1	4
		Human mastadenovirus C (Human adenovirus C)	1
		Human polyomavirus 1 (BK polyomavirus)	2
		Human polyomavirus 2	1
		Human polyomavirus 5	7
		Influenza A virus	23
		*Mycobacterium tuberculosis*	19
		Paraiso Escondido virus	1
		*Plasmodium falciparum*	1
		Primate T-lymphotropic virus 1	1
		Rhinovirus A (Human rhinovirus A)	1
		Rhinovirus C (Human rhinovirus C)	1
		SARS-CoV-2	33
		*Toxoplasma gondii*	6
		Vaccinia virus	34
		West Nile virus	6
		Yellow fever virus (Flavivirus febricis)	3
		Zaire Ebola virus	1
B*40:01	**12%** (4%)	Dengue virus	35
		Hepatitis B virus	1
		Hepatitis C virus	2
		Human herpesvirus 4 (Epstein Barr virus)	1
		Human herpesvirus 5 (Human cytomegalovirus)	4
		Human immunodeficiency virus 1	2
		Human polyomavirus 1 (BK polyomavirus)	4
		Human polyomavirus 2	3
		Influenza A virus	3
		*Mycobacterium tuberculosis*	1
		Norwalk virus (Norwalk calicivirus)	3
		Primate T-lymphotropic virus 1	5
		SARS-CoV-1	1
		SARS-CoV-2	4
B*56:01	**11%** (0.5%)	Human polyomavirus 1 (BK polyomavirus)	1
		Human polyomavirus 2	1
		*Mycobacterium tuberculosis*	1
		Yellow fever virus (Flavivirus febricis)	1
B*15:21	**11%** (0.04%)	–	–

a HLA alleles that are distinctly enriched in Indigenous Australians compared to the global population are indicated in bold type.

b Shows frequencies of the HLA allele in Indigenous Australians (ref 40) and the global estimates (ref 42).

c Indicates number of epitopes from pathogens reported in the IEDB (ref 73) for a given HLA allele. Accessed 28^th^ September 2021.

While several experimental methodologies and *in silico* prediction tools for epitope discovery have been developed, comprehensively defining novel T cell epitopes presented by the distinct and unique HLA expressed by Indigenous populations presents specific challenges. Here we review these current methodologies in the context of distinct Indigenous HLA profiles and describe in detail an immunopeptidomics approach to sequence HLA-bound peptides presented on HLA-defined virus-infected cell lines by liquid chromatography with tandem mass spectrometry (LC-MS/MS). This approach is being successfully used to comprehensively identify novel immunogenic CD8^+^ T cell epitopes for influenza A and influenza B viruses restricted by HLAs predominant in Indigenous Australians (41, 80). Given the shift in vaccine design towards targeted strategies that harness antibody and cellular immunity, understanding the T cell epitopes for HLAs representative of vulnerable Indigenous populations is critical to ensure equality of vaccine coverage and efficacy across the full diversity of global populations.

## Approaches for Epitope Identification

**Figure 2:**
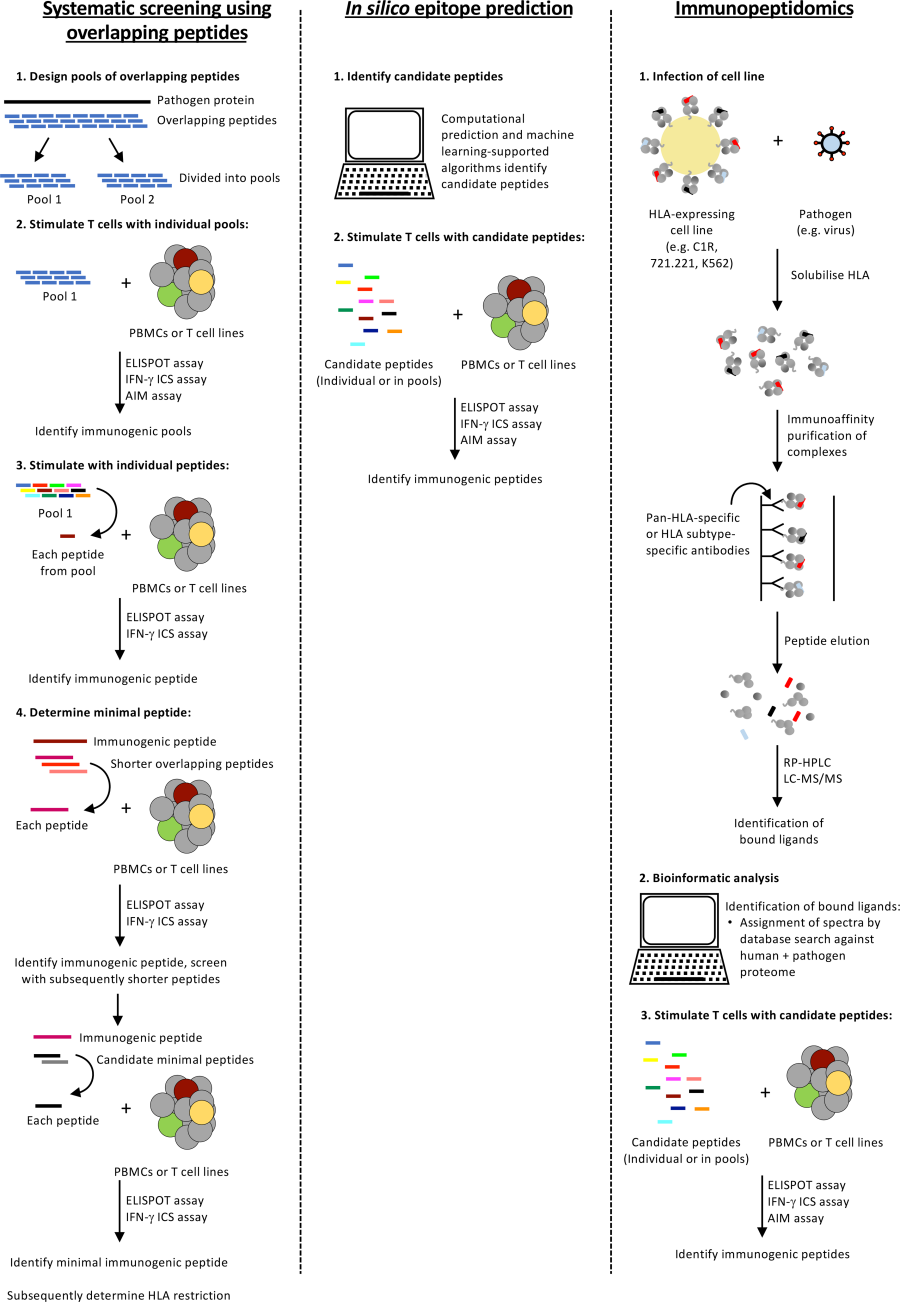
Approaches for identifying T cell epitopes for HLA alleles expressed by Indigenous populations. A summary of three epitope identification approaches.

Extensive HLA polymorphism poses a considerable challenge for epitope identification. Various experimental and *in silico* approaches (**Figure 2**) have been developed that provide a framework for dealing with the large diversity of HLA alleles (24,009 Class I HLA and 8,888 Class II HLA alleles identified as of February 2022 (IPD-IMGT/HLA Database (81)) and the array of peptides they can present. These approaches have advantages and disadvantages when considering the distinct sets of HLA alleles prevalent within Indigenous populations that are not necessarily shared at high frequency across other ethnicities (**Table 2**).

**Table 2:** Comparing epitope identification approaches, their advantages and challenges when applied to distinct sets of HLA alleles expressed by Indigenous populations.

	Systematic screening using overlapping peptides	*In silico* epitope identification	Immunopeptidomics
**Advantages**	Systematic and accurate identification of immunogenic peptides. Do not need to know donor HLA profile. Does not require specialised HLA-expressing cell lines (except if determining HLA restriction, below) or sophisticated equipment.	Rapid identification of candidate epitopes, which is advantageous in situations of newly emerged pathogens. Targeted to peptides likely to bind a given HLA, resulting in substantially reduced workload, PBMC numbers and cost compared to systematic screening. A number of prediction tools are freely available using different algorithms and prediction methods based on experimental data. Cover a wide range of HLA alleles and are continuously updated to improve predictive performance.	Identifies peptides naturally presented by a particular HLA molecule of interest/expressed HLA. Does not require prior knowledge of HLA molecules and their peptide binding preferences. Does not place assumptions on the nature of the peptides presented by HLA molecules, allowing identification of peptides with unpredictable binding modes or post-translational modifications, or from non-canonical translation products. Immunopeptidome data sets can be used to generate and improve HLA binding predictions. Data can be reanalysed by alternative bioinformatic workflows guided by new knowledge of antigen processing or the biological system to identify new ligands.
**Challenges**	Lengthy and time consuming. Requires large number of peptides (costly). Requires high numbers of PBMCs for immunogenicity screening. Challenging when limited to small blood collection volumes (30 - 70 ml) and rare donor samples. Must consider antigenic variation when selecting peptides for screening. Misses peptides that require post-translational modification. HLA restriction needs to be confirmed experimentally either using HLA-specific blocking antibodies, partially matched/mismatched cell lines or single HLA expressing cell lines. Often relies on *in silico* epitope prediction algorithms to help predict minimal peptides and/or HLA restriction.	Immunogenicity and HLA-specificity still need to be experimentally determined using HLA typed PBMCs from donors to confirm *bona fide* epitopes. Characterised by high false positive rates as predictions are heavily based on HLA binding, which does not guarantee T cell recognition. Only provide most accurate binding predictions for HLA alleles that are well-characterised. Accuracy is reduced for many rare and less-studied HLA alleles found in Indigenous populations (e.g. Indigenous Australians). May miss peptides with unpredictable binding modes or that include post-translational modification.	Requires specialized equipment (instrumentation, cell lines, software) Immunogenicity still needs to be experimentally determined using HLA typed PBMCs from donors. Requires careful development of infection models, HLA expressing cell lines and workflow for immunopeptidome analysis. Certain peptides may be lost during sample preparation and LC-MS steps due to their low abundance or chemical properties.
**Requirements**	Equipment: Cell culture equipment and reagents. ELISpot or IFN-γ ICS screening assay equipment and reagents Large numbers of PBMCs as responder cells Specialist expertise: Proficiency in cell culture and assays.	Equipment: Cell culture equipment and reagents. ELISpot or IFN-γ ICS screening assay equipment and reagents Smaller amount of PBMCs Specialist expertise: Set-up: Highly skilled expert for artificial neuronal networks For end user: Proficiency in cell culture and assays. Basic computer skills	Equipment: *(see Purcell et al. (ref 82))* Cell culture equipment and reagents. Sample preparation: cryo mill, ultracentrifuge, HLA specific antibodies, protein A/G resin MS analysis: HPLC system and separation columns, vacuum concentrator, LC-MS/MS system, bioinformatic software Specialist expertise: Cell culture and virus handling Sample preparation for MS MS acquisition and data analysis

## Systematic Screening Using Overlapping Peptides

**Figure 3:**
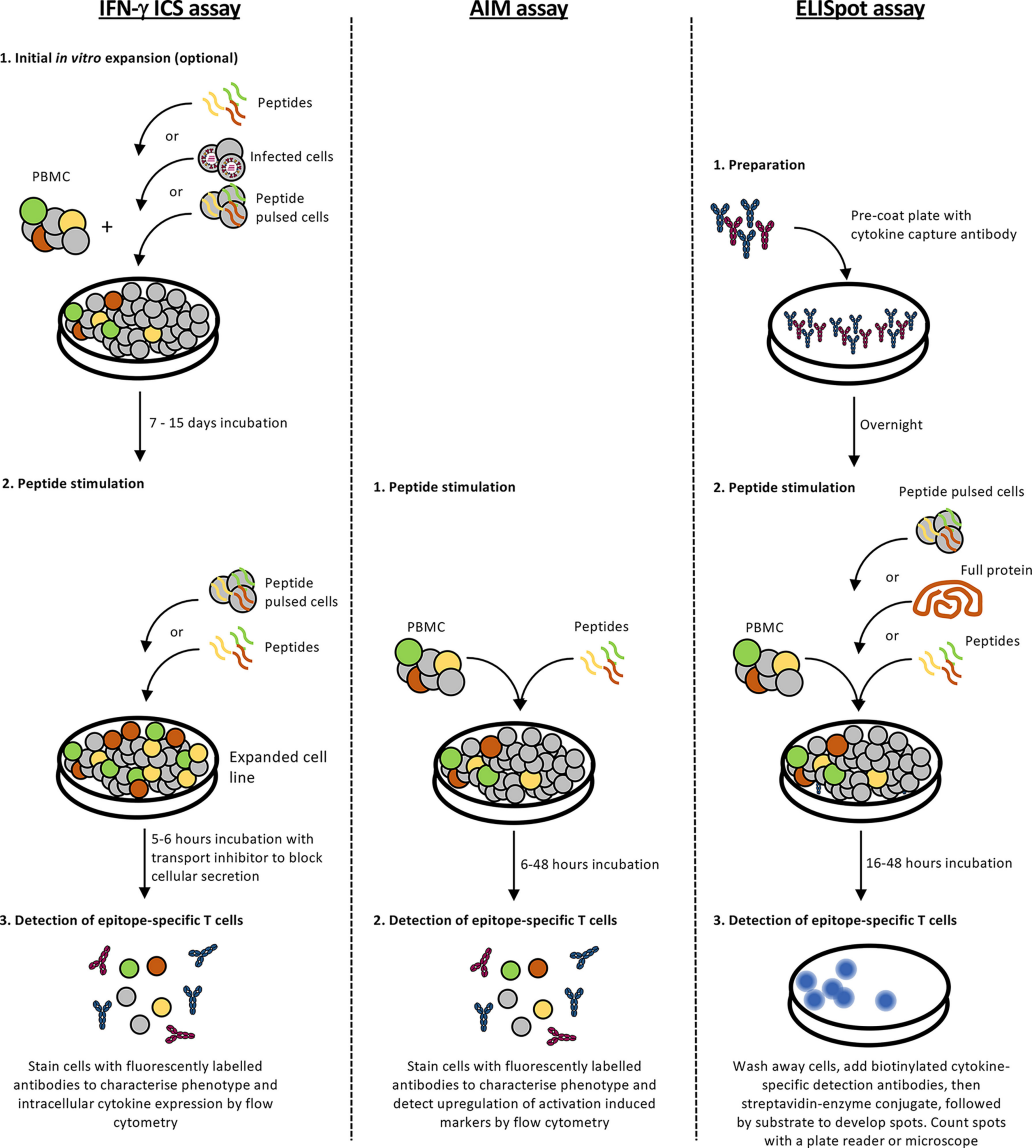
Screening for immunogenic epitopes. A summary of enzyme-linked immunospot (ELISpot), intracellular cytokine staining (ICS) and activation-induced marker (AIM) assay protocols for validating immunogenic epitopes.

A robust and accurate approach for epitope identification is systematic screening for epitope-specific T cell responses by stimulating peripheral blood mononuclear cells (PBMCs) with pools of overlapping peptides (**Figure 2**). Whilst 15- or 18-mer peptides (overlapping by 10 or 12 amino acids (aa), with 5 or 6 aa shifts, respectively) are often used, Class HLA I molecules prefer shorter peptides (8-10 aa) due to the closed ends of the peptide binding groove and consequently, screening with overlong peptides can miss responses (83). Thus, depending on the budget and responder cell availability larger pools of shorter peptides (10 aa) are often used. T cell responses to peptide pools can be characterised directly following *in vitro* peptide stimulation by detection of cytokine (ELISpot assay or intracellular cytokine staining (ICS)), degranulation (CD107a) or upregulation of specific activation-induced markers (activation induced marker (AIM) assay) (**Figure 3** and **Table 3**). Notably, the readout of cytokine assays (e.g. IFN-γ, TNF, IL-2 IL-4, IL-5, IL-9 and/or IL-17 production) needs to be tailored to the pathophysiologic state under study (84, 85). If only small numbers of PBMCs are available or the epitope-specific T cells are rare, responses can be characterised after initial *in vitro* expansion by co-culturing the PBMCs for 10-15 days with matched PBMCs pulsed with the overlapping peptide pools or infected with the pathogen of interest (86). Notably, when choosing to expand epitope-specific T cells using pathogen infection, it is important to consider the possible implications of the infection model and immunodominance hierarchies on the responses measured (e.g. the timing of protein/peptide expression, efficiency of epitope presentation in the cell line infected, epitope abundance and co-expressed HLA alleles). Expanded T cell lines are then screened for responses to the pools of overlapping peptides as above and immunogenic pools identified. The same T cell lines or PBMCs are then stimulated with individual peptides within the immunogenic pools to identify immunogenic peptides, most often using an IFN-γ ICS assay to define the peptide-specificity and CD4^+^ or CD8^+^ phenotype of the T cell response. Once an immunogenic peptide is identified, shorter overlapping peptides spanning the immunogenic peptide are synthesised (e.g. 13-mer peptides overlapping by 11 aa with 2 aa shifts) and tested for responses to determine the minimal immunogenic peptide sequence. In some cases, it may be possible to use algorithms to help predict the minimal peptide sequence, however, this relies on knowing the HLA profile of the donor and the availability of reliable data to predict the binding preferences of these HLA, something that is often lacking for the distinct HLA alleles expressed in Indigenous populations.

**Table 3:** Comparing assays used to measure T cell responses to candidate epitopes.

	IFN-γ ICS assay	AIM assay	ELISpot
**Advantages**	Detects epitope-specific cells based on expression of effector function (i.e. production of effector molecules) in response to peptide stimulation. Sensitive and high throughput. Can be performed using PBMCs directly *ex vivo*, but more commonly following *in vitro* expansion. Can combine with phenotypic markers (e.g. CD4 and CD8) to further characterise the T cell response. Permits assessment of response quality by measuring expression of multiple functions (multifunctionality). Can also compare relative amounts of effector molecule expression. Stimulation with peptide pools, then individual peptides allows identification of individual immunogenic epitopes.	Detects epitope-specific cells based on upregulation of activation induced markers in response to peptide stimulation. Sensitive and high throughput. Can use PBMCs or whole blood directly *ex vivo*. Requires fewer cells than the other assays and can be performed without *in vitro* expansion. Peptide pools commercially available. Not reliant on the expression of a particular function. Can combine a variety of activation and phenotypic markers to characterise the T cell response.	Detects epitope-specific cells based on secretion of an effector molecule (e.g. IFN-γ) in response to peptide stimulation. High sensitivity and throughput. Allows identification of rare populations. Can be performed using PBMCs directly *ex vivo* or following *in vitro* expansion. Rapid data acquisition via plate reader. Peptide pools and whole proteins commercially available.
**Challenges**	Often requires initial *in vitro* expansion over 7-13 days to increase the frequency of epitope-specific cells and facilitate their detection. As a result, the whole procedure is lengthy and time consuming. The use of *in vitro* expansion makes it difficult to relate responses back to the *ex vivo* situation. Namely, expansion will change expression of some phenotypic markers (e.g. CD27 and CD45RA) and differences in T cell expansion rates may skew frequencies of peptide-responding cells. Therefore, frequencies of epitope-specific cells detected after *in vitro* expansion may not reflect *ex vivo* frequencies or be comparable between donors. Underestimates the total epitope-specific response. Misses T cells that lack the assayed function.	Frequencies of epitope-specific T cell detected can be skewed (overestimated) by potential bystander activation. When performed on cells directly *ex vivo* it is best to screen with peptide pools that contain several epitopes or single epitopes where frequencies of responding T cells are high to enhance detection.	Only investigates the assayed function. Phenotype and polyfunctionality need to be separately investigated. Underestimates the total epitope-specific response. Misses T cells that lack the assayed function.

Determination of HLA restriction is an important step in characterising novel immunogenic peptides. As mentioned above, in some cases this can be predicted using algorithms trained to identify characteristic HLA-binding motifs or determined experimentally using antibodies that bind to specific HLA, blocking peptide presentation and T cell recognition. Alternatively, partially HLA matched/mismatched cell lines (or PBMCs) or a panel of single HLA transfected cell lines can be used as antigen presenting cells in a peptide stimulation IFN-γ ICS assay with the peptide-specific T cells to determine whether a peptide can be presented or not, allowing HLA restriction to be identified (86). These approaches are not always useful in the context of distinct or rare HLA alleles from Indigenous populations due to a lack of training data to boost the predictive power of algorithms, and lack of specific HLA blocking antibodies and suitable cell lines expressing these particular HLA.

While the systematic screening approach is lengthy, time consuming and involves large numbers of peptides which can be costly (~$50-100 per commercially synthesised peptide), it can allow for the accurate identification of immunogenic peptides independently of knowing the donor HLA profile and the peptide binding characteristics of the restricting HLA. However, reasonably high numbers of PBMCs are needed to systematically screen the full length of multiple viral proteins. Buffy coats obtained from routine blood donations (~470 ml whole blood) provide large numbers of PBMCs (>1-2x10^8^ cells), enabling epitope screening across multiple peptide pools containing relatively small numbers of peptides (10-30 peptides) (86). Meanwhile, the study of Indigenous HLA alleles requires PBMCs isolated from Indigenous individuals, which in our experience come from smaller volumes of blood (30-70 ml) kindly donated by consenting study participants. Thus, sample cell numbers (<2x10^7^ cells) become the major limiting factor for screening multiple peptide pools in Indigenous donors. Furthermore, our study of Indigenous Australians identified two new HLA alleles (HLA-A*02:741 and HLA-A*56:56) that, to our knowledge, have not been found expressed in any other ethnic group worldwide (40). These, along with other previously described unique Indigenous Australian alleles (74) occur at very low frequency (<1% Indigenous Australians), meaning that few donors with these HLA alleles are available to study, necessitating a more targeted approach. Another challenge that must be considered across all epitope identification approaches, but especially the systematic screening approach, is antigenic variation across different virus strains and lineages that may influence the detection of immunogenic epitopes depending on patterns of virus circulation and exposure history. As antigenic variation can occur particularly in epitope regions under immune pressure, leading to evasion of existing immune responses, it is necessary to consider the particular peptide sequences selected for epitope screening and potentially include pools of variant peptides to ensure responses to variable epitopes are not overlooked. For instance with the immunodominant HLA-B*07/B*35:01-restricted NP_418-426_ epitope, sequential viral variation has generated over 20 different peptide variants at TCR contact sites, some of which prime distinct CD8^+^ T cells with variable patterns of cross-reactivity to other variants (87).

Overall, systematic screening using overlapping peptides across multiple virus proteins offers the advantages of being systematic and accurate without the need for donor HLA typing or specialised equipment (laboratory equipment, engineered cell lines or software) (**Table 2**). However, is not feasible for defining T cell epitopes restricted by many of the distinct and rare HLAs found in Indigenous populations, mainly due to the high numbers of PBMCs and peptides required for thorough screening.

## *In Silico* Epitope Prediction

The threat of unpredictable novel respiratory viruses, highlighted by SARS-CoV-2, underscores the need for more accelerated methods of epitope discovery that facilitate rapid vaccine design and evaluation. The development of *in silico* epitope prediction tools enables identification of candidate peptides using computational prediction and machine learning-supported algorithms (**Figure 2**). Candidate peptides can then be screened for immunogenicity by stimulating PBMCs from suitable donors with the peptide and measuring epitope-specific T cell responses either by ELISpot, ICS or AIM assay (**Figure 3**). As HLA binding is a prerequisite for a peptide to be recognised as an epitope, *in silico* epitope prediction tools select peptide candidates based mainly on predicted HLA binding affinity which is determined through evaluation of the amino acid sequence of the peptide to forecast its compatibility for binding HLA. Such forecasting is possible because the structural and chemical properties of the HLA peptide-binding groove dictate the selective binding of peptides with compatible amino acid features. Various tools are available that differ in the experimental data or ‘training data sets’ used to develop algorithms and their methods of calculation. Training data sets generally consist of experimental data obtained from MHC binding assays measuring binding affinities of peptides to specific MHC molecules, or MHC elution data from sets of naturally processed MHC ligands eluted from cell surface MHC molecules and identified by mass spectrometry. More recent prediction tools such as NetMHCpan-4.1 and NetMHCIIpan-4.1 combine both data sets to improve prediction performance (88). MHC binding can be predicted using one of two approaches. Systems using linear regression such as PickPocket, SMM and TEPITOPE apply a fitted weight matrix to calculate a binding affinity value to a selected peptide sequence (89). The current state-of the-art methods NetMHCpan-4.0, NetMHCIIpan-4.0, MHCflurry and NNAlign use artificial neural networks to simulate MHC-binding with a variety of extra features in addition to the input peptide sequence and also allow for calculation of binding-affinity for untrained HLA alleles by comparison with closest neighbouring MHC (89). Comparison of these algorithms revealed the superiority of artificial neural network-based approaches in predicting MHC binding, but lower correlations of predicted versus measured binding affinities for strong binding peptides (89). Some algorithms such as NetChop and NetCTL-1.2 can improve epitope predictions by not only predicting the binding affinity of peptides trained on endogenously presented peptide data sets and binding affinities, but also the likelihood of cleavage by the proteasome and transport by TAP (transporter associated with antigen processing) into the ER to predict immunogenic CD8^+^ T cell targets (90). Computational tools can also be used to further refine predicted epitopes based on their potential immunogenicity, robustness, allergenicity, toxicity and autoimmunity. One of these algorithms, NetCTLpan showed up to 40% reduced experimental workload to identify 90% of new epitopes compared to prediction tools that only assess MHC binding affinity (90).

*In silico* epitope prediction is of high interest for quickly identifying T cell epitopes when new pathogens emerge, speeding up identification of novel T cell targets that allow for the assessment of immune responses or the development of T cell activating vaccines. These tools were utilized for example to efficiently predict epitopes for various HLAs during the COVID-19 pandemic, highlighting their emerging importance in research (see below). Another situation where epitope prediction tools are superior to classical screening is in personalized cancer treatment. Cancer cells can be sequenced and neo-epitopes predicted for use in a vaccine to induce cancer-specific T cells. This allows a level of personalized treatment that was unachievable previously and is already efficiently used in clinical trials. BioNTech was able to show that vaccination with personalised melanoma neo-epitopes could induce T cell responses in all participants and resulted in killing of neo-epitope-expressing melanoma cells (91). Likewise, Ott et al. (92) demonstrated induction of highly specific, polyfunctional CD4^+^ and CD8^+^ T cell responses that targeted a broader range of neo-epitopes than induced by existing immunotherapeutics and prevented disease recurrence in 4/6 patients up to 25 months post-vaccination. Thus, aided by epitope prediction tools, this treatment potentially overcomes key challenges to effective cancer therapy including individual tumour heterogeneity, selective targeting of tumour versus healthy tissue and tumour escape through loss of antigen. An intriguing idea is that computation tools could potentially be adapted to use self-peptidome data to establish rules that predict peptides to be avoided for use for infectious disease or cancer indications, perhaps as they have motifs or properties that are too similar to self and potentially could cause autoimmunity. Lastly, the usage of epitope prediction tools can substantially reduce workload and cost by limiting the amount of peptides required to screen, an attractive feature in the context of rare and limited donor samples.

*In silico* epitope prediction methods are characterised by high false positive rates, in that many of the predicted epitopes are found to be non-immunogenic. For example, Zheng and colleagues used *in silico* analysis to predict novel hepatitis B virus polymerase epitopes for the most common human MHC-I, HLA-A*02:01 (93). Of the pre-selected epitopes, 25% (2 out of 8) demonstrated good ability to induce immune responses and suppress hepatitis B virus replication *in vitro.* In a different study, only 13 out of 225 (6%) predicted tumour-associated antigens showed immunogenicity in an *in vivo* experiment using transgenic mice (94). The efficiency of *in silico* epitope prediction can depend on factors such as the protein sequence analysed, the MHC molecule of interest and the data set used for training. However, a key limitation of the algorithms is that they predict HLA binding as a surrogate for epitope presentation, which does not guarantee T cell recognition and activation, i.e. immunogenicity. Future algorithms could potentially be enhanced by incorporating information on potential T cell receptor (TCR) interactions as well as comparison to other endogenously presented peptides on which the immune system is trained. Inclusion of these parameters could increase the frequency of correctly predicted immunogenic T cell epitopes.

A further limitation is that most *in silico* epitope prediction studies and experimental validations are biased towards certain HLA alleles. All methods of *in silico* prediction rely on previously published data and, as already outlined, the identification and study of T cell epitopes often focuses on common and widely-expressed HLA alleles. As such, these algorithms are constrained by the training data and can therefore only provide most accurate guidance for MHC alleles that are well-characterised or similar to well-characterised MHCs (95, 96). Their accuracy is reduced for rare or less-studied HLA alleles that are poorly represented in databases, which is the case for certain HLA alleles found in Indigenous Australians. Inclusion of additional HLA-specific binding and elution data sets is needed to further improve the capacity of these algorithms to accurately predict T cell epitopes for Indigenous populations.

While initial programming of artificial neural networks and generation of training data is both expensive as well as requiring highly trained personnel, if well programmed the resulting epitope prediction tools are extremely easy to use for the end user (**Table 2**). They significantly reduce the budget required to purchase and screen candidate peptides, facilitating research on smaller populations like Indigenous Australians or less common pathogens that might not be of high commercial interest for bigger pharma companies or attract sufficient funding for high-scale experiments.

## *In Silico* Epitope Prediction and COVID-19

*In silico* epitope prediction is highly advantageous in situations of newly emerged pathogens, utilizing previously determined peptide data sets and the novel pathogen’s genetic sequence. Shortly after the genetic sequence of SARS-CoV-2 was published in early 2020 (97), *in silico* methods were used to predict T cell epitopes as potential targets for SARS-CoV-2 vaccines. Two rationales for *in silico* prediction have been used to predict SARS-CoV-2 epitopes, i) utilizing the genetic similarities between SARS-CoV-1 and SARS-CoV-2 and ii) peptide-HLA binding prediction methods. Epitopes derived from SARS-CoV-1 structural proteins induce long lasting T cell immunity (98, 99) and these proteins share high genetic similarity with SARS-CoV-2, ~76% for spike and >90% for nucleoprotein, membrane, and envelope proteins (100). The high genetic similarity prompted *in silico* studies to use T cell epitopes from SARS-CoV-1 immunological studies to predict likely SARS-CoV-2 T cell targets (98, 100–103). As sequencing data from new SARS-CoV-2 variants continued to emerge, a web-based platform, COVIDep, was developed to enable the identification of experimentally-derived SARS-CoV-1 epitopes that have a close genetic match with the latest available sequencing data (104). Nonetheless, the large majority of *in silico* SARS-CoV-2 studies have predicted T cell epitopes using existing peptide-HLA binding prediction methods, which benefit from years of peptide-HLA research. *In silico* predicted epitopes have been derived from all 12 SARS-CoV-2 proteins, with the spike protein being the most analysed protein, though unsurprisingly, there is considerable overlap among the sets of epitopes for SARS-CoV-2 predicted using different methods. As of September 2020, 2,239 HLA class I and 2,580 HLA class II epitopes had been predicted using *in silico* methods for SARS-CoV-2 (105). However, a low proportion of these have been experimentally validated, reflecting a disconnect between *in silico* studies and validation studies. Of interest, a study focusing on South American populations used updated HLA frequencies and *in silico* epitope prediction tools to establish a tailored list of 27 HLA-I and 34 HLA-II candidate epitopes that would provide high regional coverage if incorporated in a vaccine against SARS-CoV-2 (106). These candidates achieved better regional coverage than other approaches that aimed to identify epitopes that cover the global population. This highlights the strength of epitope prediction tools to rapidly identify candidate epitopes for a defined HLA profile. However, it also shows how approaches based on broad population coverage can result in reduced regional coverage due to the associated HLA profile.

While *in silico* epitope prediction is incredibly effective at generating large databases of potential epitopes, immunogenicity and HLA-specificity need to be experimentally determined to confirm bona fide epitopes. PBMCs from individuals recovered from COVID-19 have been used to experimentally characterise *in silico* predicted peptides. T cell activation can be measured following *in vitro* peptide stimulation (ELISpot, ICS or AIM assay, see below) either directly (102, 107, 108) or after initial *in vitro* expansion by co-culturing (109–111). Additionally, HLA restrictions of the reported epitopes can be experimentally determined by using multimer qualitative binding (112–114). Following experimental validation, over 700 unique SARS-CoV-2 T cell epitopes with known HLA allele restriction have been reported, including 20 immunoprevalent epitopes (>50% of tested recovered individuals responded) registered across multiple cohorts (115). This is highlighted by the experimental-verification of peptide-HLA binding predicted epitopes restricted to HLA-A*02:01 and HLA-A*24:02. From epitopes predicted by NetCTLpan and/or NetMHCpan, three HLA-A*02:01 (S_269–277_, S_976–984_ and Orf1ab_3183–3191_) and three HLA-A*24:02 (S_1208–1218_, S_448–456_ and S_193–201_) epitopes were found to be immunogenic following *in vitro* expansion of PBMCs from COVID-19 donors for 10-12 days (110, 111). Importantly, while these HLA alleles are common and widely-expressed globally, they are also highly expressed in Indigenous Australians. Following identification of novel SARS-CoV-2 epitopes, T cells tested directly *ex vivo* from COVID-19 patients and pre-pandemic healthy individuals can be assessed for their phenotype, function and TCR. Different SARS-CoV-2 epitopes have been found to have different immunodominances, with A1/ORF1a_1637_ and B7/N_107_ currently identified at higher precursor frequencies compared to the known HLA-A*02:01 and A*24:02 epitopes (112, 114, 115). T cells specific to SARS-CoV-2 epitopes restricted to both HLA-A*02:01 and A*24:02 have a predominantly naïve phenotype in pre-pandemic individuals, whereas the phenotypic profile in COVID-19 donors is more varied with skewing towards memory phenotypes (111, 112). The clonal composition and diversity of the TCR repertoire can impact immunodominance, functionality and protection. Recent studies have suggested that TCRαβ diversity might be linked with the prominence of SARS-CoV-2 CD8^+^ T-cell responses during the primary infection. The subdominant A2/S_269_^+^ and A24/S_448_^+^ CD8^+^ TCRαβ repertoires were driven by restricted motifs, whereas the A1/ORF1a_1637_^+^CD8^+^, B7/N_105_^+^CD8^+^ and A24/S_1208_^+^CD8^+^ TCRαβ repertoires were more diverse across COVID-19 patients (111, 112, 116).

Characterization of SARS-CoV-2 T cell epitopes is important in the fight against COVID-19 and for protecting Indigenous populations. *In silico* epitope prediction and experimental verification of SARS-CoV-2 T cell responses has assembled a data set of key T cell epitopes presented by a range of HLA alleles. Analysis of epitopes for mutations in SARS-CoV-2 variants of concern can help with monitoring potential viral escape from T cell responses, with the identification of epitopes capable of eliciting broadly cross-reactive immunity across emerging variants of upmost importance. Furthermore, novel antiviral vaccines developed for COVID-19 can elicit both antibody and T cells responses (117, 118). Therefore, the discovery of SARS-CoV-2 epitopes allows epitope-specific T cells to be tracked and correlates of SARS-CoV-2 protection defined following infection and vaccination. Finally, the identification of immunogenic T cell epitopes could aid the rational design and evaluation of the next-generation universal COVID-19 vaccines, especially for high-risk groups such as Indigenous people.

## Immunopeptidomics for Identification of Candidate T Cell Epitopes

Mass spectrometry-based characterisation of the peptides presented by HLA/MHC molecules, or immunopeptidomics, has come to the forefront in the last 10 years as a means to detect naturally processed and presented peptide ligands. The great benefit of these analyses is that they do not place assumptions on the nature of the peptides that can be liberated through enzymatic processing, nor do they rely on sufficient knowledge of the interrogated HLA/MHC molecules for accurate binding predictions. Not only does this allow for identification of ligands that adopt unpredictable binding modes (e.g. overhanging/extended HLA-I ligands (119–121)), but it also allows detection of post-translationally modified peptides of biological relevance (e.g. glycosylation, deamidation, phosphorylation) as well as those from non-canonical sources (alternative reading frames, UTRs) that would not necessarily be considered when designing overlapping peptide libraries or performing binding predictions.

HLA-I molecules constitutively present peptides derived from the enzymatic processing of proteins produced within the cell. Characterisation of these peptides *via* mass spectrometry, especially from cell lines engineered to express single HLA-I alleles, has been used to help define peptide binding preferences of numerous HLA variants (121–126). However, in the context of viral infection, immunopeptidomics also represents a method for the identification of naturally presented candidate epitopes for subsequent immunogenicity screening (for example (41, 127–130)). In-depth descriptions of the methodologies for immunopeptidome analysis can be found elsewhere (82, 131). The basic process (**Figure 2**) generally involves:

1. Solubilisation of the MHC/HLA from cellular material *via* non-denaturing lysis to maintain complex conformation. Alternatively, cells can be engineered to express soluble forms of MHC/HLA which can be harvested from cell culture supernatant (125, 126, 128, 129, 132), while soluble HLA can also be isolated from blood serum (133, 134).
2. Immunoaffinity purification of complexes using either pan-specific (e.g. W6/32 for all HLA-I) or sub-type specific (e.g. BB7.2 for HLA-A2) HLA/MHC antibodies.
3. Peptide elution *via* denaturation (usually acid elution).
4. Removal of HLA/MHC heavy and light chains. For example, *via* reversed-phase high performance liquid chromatography (RP-HPLC) (including fractionation the peptide ligands) or using molecular weight cut-off filters.
5. Liquid chromatography-tandem mass spectrometry (LC-MS/MS).
6. Bioinformatic analysis.

Workflow modifications can be incorporated to increase robustness of quantitative comparisons (e.g. targeted analysis using Multiple Reaction Monitoring) (135), to enrich for specific modifications within the sample such as phosphorylation (136), or to interrogate samples for specific post-translational modifications (e.g. spliced peptides, glycosylation) (137, 138). Currently (February, 2022) more than 970,000 MHC ligands have been collated within the IEDB (73) that have been identified using mass spectrometry (Search filters: Include positive Assays, MHC Assays : Ligand presentation|mass spectrometry, ligand presentation|cellular MHC/mass spectrometry, ligand presentation|secreted MHC/mass spectrometry). Amongst these are more than 3000 (non-redundant by sequence and modification) derived from viruses including vaccinia (>900), SARS-CoV-2 (>800), and influenza viruses (>700).

As mentioned above, screening of large numbers of peptide pools is often prohibitive due to low sample volumes available for immunogenicity screening. An immunopeptidomics approach to sequence HLA-bound peptides by LC-MS/MS generates a more highly curated and biologically relevant list of peptides to screen *via* ELISpot, ICS or AIM assays compared to overlapping peptide libraries or binding predictions alone. Applying this approach to specific HLAs relevant to Indigenous populations enables comprehensive analyses of peptide presentation regardless of how well-studied the HLA allele is or the availability of relevant PBMCs. Nevertheless, whilst an in-depth mass-spectrometry-based immunopeptidomics approach is very successful at defining large numbers of peptides presented by HLA/MHC molecules during infection (41, 80, 119, 127, 130), many peptides eluted from HLA molecules are not immunogenic. For instance, in our immunopeptidomic study of HLA-A*24:02, dominant responses were detected against 3-4 (depending on ethnicity) out of 54 IAV peptides tested, and 6 out of 41 IBV peptides tested (41) (data from peptide elution studies are available through IEDB (http://www.iedb.org/refId/1039181) and the ProteomeXchange Consortium *via* the PRIDE (139) partner repository (accession PXD020292)). However, another study of ~170 vaccinia virus pMHCI presented on infected mouse cells found nearly 40% were immunogenic in more than half of the C57BL/6 mice screened (140). This again reflects the fact that HLA binding is necessary but not sufficient for immunogenicity and screening peptides identified using an immunopeptidomics approach with relevant PBMC samples will always be necessary to identify immunologically relevant T cell epitopes.

While LC-MS/MS-based analysis of the immunopeptidome can achieve identification of many thousands of peptides in a single experiment (including a subset of viral peptides in infection experiments), certain limitations and challenges should be acknowledged. Firstly, relatively large cell numbers are required for broad coverage of the immunopeptidome due to the overall low abundance of many bound species. Secondly, certain peptides may be lost during sample preparation due to lack of binding to chromatographic stationary phases (e.g. highly hydrophilic peptides may display low interaction with the stationary phase used during RP-HPLC separation, which can be further exacerbated by oxidation at methionine residues), while highly hydrophobic peptides (e.g. the immunodominant HLA-A*02:01-restricted IAV epitope M1_58-66_ GILGFVFTL) may display low solubility in aqueous buffers. Thirdly, MHC-bound peptides which lack positively charged residues may exist as singly-charged ions and are often excluded from fragmentation in traditional proteome analysis based LC-MS methods which are deliberately biased to charge states of 2 or greater (typical of tryptic peptides which terminate in positively charged residues) to avoid fragmentation of singly charged contaminants common in mass spectrometry analysis. Inclusion of singly charged species has been shown to increase the number of MHC ligands identified for certain HLA alleles, although not incorporated by all studies (131). Indeed, differences in preparative, analytical and bioinformatic workflows have been shown to impact the properties of ligands identified by immunopeptidomics, with different pipelines inducing biases for/against specific amino acids (e.g., hydrophobic/basic/proline) that should be considered during workflow development (141, 142). Moreover, these workflows are susceptible to contamination by non-HLA bound protein fragments. Possible contaminants can be highlighted bioinformatically using strategies including GibbsCluster 2.0 (to identify outliers) (143), assessment of peptide ladders generated by proteolytic degradation (144) or comparison with similar data sets for common contaminants. Ultimately however, when searching for novel epitopes, application of immunopeptidome analysis for epitope discovery can be considered hypothesis generation, with immunogenicity testing as the final validation of biological relevance.

Finally, compared to using overlapping peptide libraries or performing binding predictions, immunopeptidome analysis requires access to specific materials such as appropriate cell lines, HLA-specific antibodies and immunoaffinity purification reagents, as well as instrumentation including a HPLC system, vacuum concentrator and high resolution LC-MS/MS. Furthermore, specialist expertise in sample preparation for MS analysis to avoid introduction of problematic contaminants, LC-MS/MS acquisition and bioinformatic analysis, as well as initial infection protocols are also required (**Table 2**).

Although we have stated that one of the great strengths of immunopeptidome analysis is detection of naturally processed and presented peptides that might be missed by binding predictions, immunopeptidome data sets can also be used to generate and improve MHC/HLA binding predictions. Indeed, as mentioned above, prediction tools such as NetMHCpan and HLAthena preferentially or even exclusively incorporate elution data to train their prediction algorithms (88, 123), while others seek to enable input of user defined data sets to generate bespoke MHC/HLA binding predictions (145). In the context of understudied HLA/MHC with limited or no published ligands/affinity data/immunopeptidome data, such as several of those expressed by Indigenous Australians, new immunopeptidome data sets represent a critical avenue to generate and improve HLA-binding predictions. Thus, in-depth immunopeptidomic investigation of a single infection model in the context of an understudied HLA, while directly detecting potential epitopes for downstream analyses, can also be repurposed, utilising the large data sets of host and pathogen derived peptide ligands to train binding models for application to alternate pathogens which are not as amenable to analysis (e.g. high biosafety level, poor infection efficiency).

## Considerations for Single HLA Analysis *via* an Immunopeptidomics Approach

When available, the use of HLA-specific antibodies for isolation of peptide-bound HLA-I molecules can enhance the accuracy of assigning HLA binding (for example we utilised the anti-HLA-A2 antibody BB7.2 to specifically isolate HLA-A*02:01 from the C1R in our study of IBV peptide presentation (127)), which is of greater importance against more complex HLA backgrounds. However specific antibodies are not available for most HLA alleles. Normal human cell lines can express up to 6 distinct classical HLA-I at the cell surface due to HLA co-expression and heterozygosity. This generates a mix of HLA binding specificities when performing isolations with pan HLA-I antibodies. Several bioinformatic approaches have been generated to deconvolute multiple HLA-binding motifs from multi-allele data sets and represent one avenue to distinguish individual binding specificities (e.g (143, 146, 147)). Alternatively, single HLA-expressing cell lines with no or low endogenous HLA simplify this problem and facilitate the assignment of peptide ligands to a specific HLA allomorph with less bioinformatic processing. Single HLA-expressing cell lines are often generated *via* electroporation with a plasmid encoding the HLA of interest and cultured under conditions of antibiotic selection to maintain stable transfectants (148), or more recently by adapting retroviral transduction protocols (149) to stably transduce HLA genes (150) (L. Hensen, E. B. Clemens and K. Kedzierska, unpublished protocol. See reagent availability note.). Classical HLA-I-reduced (e.g. C1R) or null (721.221, K562) cell lines are valuable tools for both characterisation of peptides presented by a transfected HLA of interest and functional dissection of T cell epitope restriction (122–124). C1R cells are derived from EBV-transformed B cells passaged after three rounds of γ-irradiation and immunoselection for reduced HLA-I expression (151). C1R cells do not express any HLA-A molecules, have a markedly reduced expression of HLA-B*35:03 and stably express HLA-C*04:01, and our frequent use of this line in immunopeptidome characterisation has given us in-depth knowledge of the peptides presented by these endogenous HLA molecules (41, 127, 152). Permissivity to infection is an important feature of the cell line to be used and notably, while influenza virus strains differ in their ability to infect B cells (153–155), C1R cells can be readily infected with laboratory strains of IAV and IBV *in vitro* (41). Through insertion of single HLA genes into C1R cell lines, cell surface presentation of peptides by HLAs of interest can be assessed during infection without the need for HLA-I-subtype specific antibodies to enable identification of CD8^+^ T cell epitopes presented under physiological conditions of cellular virus infection. Alternatively, transfection of cells with DNA constructs to express soluble HLA, which is then harvested from the supernatant, is an alternative approach for interrogating peptides from single HLA variants (125, 126, 128, 129).

It should be acknowledged that presentation by single HLA-expressing cell lines such as C1R may not be a perfect mimic of presentation in the *in vivo* context, and may either miss epitopes generated in *in vivo* infected cell types (e.g. lung epithelia), or over-represent certain peptides. During infection *in vivo*, presentation by professional antigen presenting cells such as dendritic cells is important for T cell priming, whilst presentation by different infected cell types (e.g. epithelial cells of the airways during influenza (156) and SARS-CoV-2 (157) infection) is critical for their clearance. Altered quantitative hierarchies of epitope presentation *via* cross presentation on dendritic cells versus direct presentation on infected cells have been shown for influenza epitopes in mice, suggesting presentation pathway and presenting cell types may be factors that influence the immune response (158). Furthermore, processing enzyme expression also impacts peptide presentation with C1R cells, for instance, expressing standard and immunoproteasomal subunits (159) and specific ERAP1 and ERAP2 variants for which polymorphisms alter cleavage efficiencies (160).

During *in vivo* infection antigen presentation at different anatomical sites (e.g. the airways) is likely changeable dependent on the cellular response to infection and the inflammatory environment at different stages of infection. Inflammatory cytokines have been shown to cause marked changes in the immunopeptidome through modulation of components of antigen processing and presentation, including expression levels from different HLA loci and of the standard proteasome and immunoproteasome subunits, which can alter the levels of specific epitopes at the cell surface (161–165). Timing post-infection should also be considered, as in our own studies involving IAV and IBV infection we observed broadest peptide identification 8-12 hrs post infection (41), while down regulation of HLA molecules, a common strategy for viral evasion of cellular immune responses (166) (reviewed in (167, 168)), is observed at late stages of IAV and IBV infection *in vitro* (169), potentially impacting the breadth of peptide presentation. This may change for different viruses, strains and infection models. Thus, the development of models that more closely mimic the *in vivo* setting is of great interest (170) and a consideration for the success of this strategy. For viruses with low infection efficiency of cell lines, or for which high physical containment is required, analysis of presentation of specific viral proteins can also be performed by transfection with expression constructs for viral antigens (171). Moreover, the self-immunopeptidome of these lines can be used to build peptide binding predictions for these understudied HLA molecules, as mentioned above. These binding predictions provide a secondary lens through which to assess identified candidates to increase confidence of assignment as binders of a given HLA and differentiate contaminants. For understudied HLA allomorphs, where limited training data is available for binding predictions, the co-isolated self-derived immunopeptidome is of increased importance for understanding binding preferences and triaging candidate epitopes.

## Validation and Characterisation of T Cell Responses to Candidate Epitopes

Specific T cell responses to candidate epitopes can be measured using a variety of different assays (**Figure 3** and **Table 3**). ELISpot and IFN-γ ICS assays are sensitive and high throughput methods that measure epitope specificity based on cytokine production by T cells in response to peptide stimulation. These assays are particularly useful for screening responses initially to peptide pools, then smaller pools or individual peptides to identify minimal immunogenic epitopes. The assays can be performed using PBMCs directly *ex vivo* or following *in vitro* expansion using autologous PBMCs pulsed with peptide pools to expand epitope-specific T cells, particularly if the epitope-specific T cells of interest are present at low frequencies. Other methodologies for expansion such as virus infection or whole antigens would also be suitable. T cell lines expanded *in vitro* can then be stimulated with the corresponding peptide pool or individual peptides for detection of immunogenic peptides. The ICS assay has the advantage of discriminating CD4 and CD8 phenotype and detecting expression of multiple functional molecules (e.g. IFN-γ, TNF, MIP-1α, IL-2 and CD107a), allowing measurement of polyfunctionality and phenotypic subsetting. However, ELISpot is more sensitive at measuring low-level responses (172).

Using an ICS approach, we have recently identified human CD8^+^ T cell epitopes restricted by HLA-A*11:01 for influenza A and influenza B viruses in Indigenous and non-Indigenous people (80). HLA-A*11:01 is highly prevalent in Asian and Indigenous Australian populations, making it an ideal candidate for targeting CD8^+^ T cell immunity in a large proportion of the global population. Multiple influenza A virus CD8^+^ T cell epitopes restricted by HLA-A*11:01, or the HLA-A*03 supertype, have been reported (45, 173–178). HLA supertypes, defined by similarity in anchor pockets responsible for binding the primary anchor residues of peptide ligands, can be useful starting points for less studied alleles (179–181). Due to shared peptide binding features, HLA alleles within the same HLA supertype are likely bind similar peptides, potentially simplifying the need to perform epitope analyses for each of the >30,000 HLA allelic variants that exist (81). However, polymorphisms across the antigen binding cleft can impact both the binding and conformation of bound peptides within supertypes (182, 183), resulting in very different outcomes for T cell activation (184, 185). Thus, the idea that HLA molecules from the same supertype will bind the same peptide and activate an immune response does not always hold true (181). We endeavoured to confirm the immunogenicity of previously reported HLA-A*11:01-restricted IAV peptides and identify novel peptides using immunopeptidomics. Similar to the work on HLA-A*24:02 (41), IAV peptides were screened by pulsing HLA-A*11:01^+^ PBMCs with peptide pools containing 10 IAV peptides each, and co-culturing with unstimulated, autologous PBMCs. After culturing for 10-12 days *in vitro*, cells were restimulated with pooled or individual peptides and ICS performed to determine CD8^+^ T cell activation. Two peptide variants, PB2_320-331_ and PB2_323-331_, were found to be immunogenic in 2 of 7 individuals tested. Overall, the immunogenicity of IAV peptides restricted by HLA-A*11:01 was relatively low, suggesting that robust CD8^+^ T cell mediated immunity toward IAV may not be best directed by HLA-A*11:01. Unlike the known HLA-A*11:01-restricted IAV epitopes, influenza B epitopes have only recently been identified for HLA-A*11:01 using our immunopeptidomics approach. The immunogenicity of identified HLA-A*11:01-restricted IBV peptides was determined using an IBV infection method for T cell expansion. This involved infection of HLA-A*11:01-expressing C1R cells with B/Malaysia/2506/2004 to stimulate and expand antigen-specific CD8^+^ T cells from HLA-A*11:01-expressing individuals *in vitro* (41, 80). CD8^+^ T cells were restimulated with pooled or individual IBV peptides and their activation was determined *via* ICS. To this end, three IBV peptides were found to be immunogenic, the strongest of which was M1_41-49_, while subdominant responses were observed with NS1_186-195_ and NP_511-510_.

ICS approaches are highly useful and readily adaptable for screening and analysing T cell responses to candidate peptides. However, it is important to note that ELISpot and ICS assays can underestimate the magnitude of epitope-specific responses compared to assays that measure TCR binding to pMHC (118), presumably because some cells may lack the particular function assayed (186, 187). Furthermore, any screening strategy using PBMCs from human donors must consider variability in pathogen strains commonly circulating in the geographic regions of the donor cohort and the presence of naturally occurring peptide variants. Tools such as the Influenza Research Database “Identify short peptides in proteins” analysis tool (188) are useful to search for naturally occurring peptide variants to include in epitope mapping and assess the cross-reactivity of CD8^+^ T cell responses to epitope variants. In addition, when screening a large number of peptides, it is advantageous to arrange the peptides in pools relative to their predicted binding affinity for the HLA of interest (using a prediction tool such as NetMHCpan4.0) to potentially avoid high affinity peptides outcompeting low affinity peptides for HLA binding which might result in reduced sensitivity of the assay.

Aside from cytokine expression, epitope-specific T cells can also be detected following peptide stimulation by cell surface expression of CD107a, a marker of degranulation, or activation induced markers. Since *in vitro* culture and peptide-driven expansion can alter expression of activation, phenotypic and memory markers, an AIM assay is often performed *ex vivo* with the use of peptide pools to enhance detection. PBMCs or whole blood can be used, and cells are stimulated with peptides for 24 to 72 hrs. A variety of different markers can be assessed to determine T cell activation. The most common markers for CD4^+^ T cells include OX40 (CD134), CD25 or CD137 (189–191). Other markers that have been tested include CD69 and PD-L1 (192). These markers overlap with activation markers for CD8^+^ T cells where CD69 and CD137 have been shown to deliver robust results (190, 191). The AIM assay allows for rapid detection of epitope-specific cells and is not reliant on the expression of a particular function or proliferation in contrast to *in vitro* expansion of epitope-specific T cells, while also requiring fewer cells. However, due to the low frequency of epitope-specific T cells *ex vivo*, it is only suitable for screening for responses to bigger peptide pools that contain several epitopes or single epitopes where frequencies of epitope-specific T cells are high. It is often unsuitable for the identification of individual epitope-specific populations of T cells in donors. It is, however, very useful to compare total T cell responses towards peptide pools derived from whole proteins when epitopes or the donor HLAs are unknown, for example when comparing vaccine responses or responses induced by infection derived from novel pathogens (189, 190). In particular, assessing the total magnitude of T cell responses to vaccination may be more informative for gauging the overall robustness of vaccine responses than measuring responses to individual epitopes. Screening T cell responses across different populations could be used to determine vaccine responses in various ethnicities or identify vulnerable populations that might require modified vaccines, such as adjuvanted, high dose or inclusion of additional immune cell targets.

Snyder et al. (193) have combined the AIM assay with a prior stimulation of memory T cells using an anti-CD3 antibody for 8-13 days. This can increase the total number of available cells for screening, facilitating detection of low frequency epitope-specific populations or overcoming constraints due to limited donor blood volumes. The suitability of this protocol modification however depends on the research question. One of the advantages with the AIM assay is that the results are very representative of *ex vivo* responses. However, stimulation for at least 8 days with a stimulatory antibody will substantially alter the phenotype of the cells. Furthermore, it is unclear if different T cells expand at different rates, skewing the frequency of peptide responding cells measured, especially if they are derived from donors that had recent contact with the antigen. Unfortunately the authors do not compare the frequency of their epitope-specific populations prior to and post expansion (193). However, in the context of novel antigens, where the amount of relevant donors and samples are limited, this adaptation of the AIM assay could be extremely useful for initial screens followed by more in-depth analysis.

HLA tetramer/multimer assays detect epitope-specific T cells based on TCR binding of a given peptide-HLA complex. This approach necessitates the specific synthesis of peptide-HLA complexes of interest, hence it is not used for large scale screening of many candidate epitopes and is instead reserved for subsequent in-depth characterisation of selected epitope-specific T cells. HLA tetramer/multimer enrichment techniques can be used to enumerate and isolate low frequency epitope-specific T cells, including naïve epitope-specific T cells. The identified populations can be used for multiparameter phenotypic analysis, functional assays, TCR sequencing and transcriptomic profiling. These can provide powerful insights into the specificity, function and quality of epitope-specific CD8^+^ T cells and how these can best be harnessed for stronger, durable and cross-protective immunity.

## Approaches for Epitope Identification – Pros and Cons

In summary, systematic screening using overlapping peptides, *in silico* epitope predictions and immunopeptidome analysis each have their advantages and challenges (**Table 2**). Whilst overlapping libraries encompass the full sequence of the tested antigen, they are reagent greedy and fail to encompass post-translational modifications. Similarly, *in silico* methods rely on sufficient training data for high accuracy predictions and may miss peptides that adopt unanticipated binding modes or incorporate post-translation modifications. In contrast, generation of lists of candidate epitopes from immunopeptidome analysis focusses analyses on peptides that are naturally processed for presentation by the HLA and can encompass post-translational modifications. However this workflow may miss less abundant species, or peptide species that are lost during sample preparation due to biophysical properties that reduce interaction with chromatographic columns. Overall, whilst each technique has blind spots, they each have the capacity to reveal important epitopes to inform vaccine development, whether in the form of polyepitopes or large epitope rich portions of the target pathogen.

## Concluding Remarks

The importance of T cell epitope identification has never been more clear as it is now in the face of an ongoing COVID-19 pandemic. T cell epitope identification is highly relevant to situations where new viruses capable of infecting humans emerge or re-emerge. It is also of great importance in the context of globally diverse populations and ethnicities, including immunologically vulnerable Indigenous populations. A number of successful strategies for epitope identification are now available, but HLA alleles prominent in Indigenous populations require special consideration, because of the often distinct, rare and understudied nature of these molecules. Studies to understand the peptide repertoires and immunogenic epitopes presented by HLA alleles prevalent in Indigenous people provide vital insights that inform the rational design and evaluation of T cell-based vaccines to ensure they provide Indigenous populations worldwide with effective protection from infectious diseases.

## Reagent Availability

C1R cell lines expressing key Indigenous Australian HLA (HLA-A*11:01 (80), 24:06, 24:13, 34:01, B*13:01, 15:21 (150), 15:25 (150), 40:01, 40:02, 56:01, 56:02, 56:56) are available upon requestion from KK. Requires a materials transfer agreement (MTA) from The University of Melbourne.

## Author Contributions

EC, KK, LH, PI and AP planned the manuscript. EC, KK, LH, PI, LR and JH wrote sections of the manuscript and generated figures. JD, AM, ST, CS, KF and AP contributed specialist expertise, perspectives and data for the manuscript and contributed to modification of the original and revised versions of the manuscript. EC, KK, LH, PI and AP wrote and modified the original manuscript and the revised versions.

## Conflict of Interest

The authors declare that the research was conducted in the absence of any commercial or financial relationships that could be construed as a potential conflict of interest.

## Publisher’s Note

All claims expressed in this article are solely those of the authors and do not necessarily represent those of their affiliated organizations, or those of the publisher, the editors and the reviewers. Any product that may be evaluated in this article, or claim that may be made by its manufacturer, is not guaranteed or endorsed by the publisher.
